# Protocol for the Paediatric Otorrhoea Study (POSt): a multi-methods study to understand the burden of paediatric otorrhoea in the UK

**DOI:** 10.1136/bmjopen-2023-078052

**Published:** 2023-09-05

**Authors:** Elliot Heward, James Dempsey, Judith Lunn, John Molloy, Rachel Isba, Matthew Carr, Darren Ashcroft, Alastair D Hay, Jaya R Nichani, Iain A Bruce

**Affiliations:** 1 Division of Infection, Immunity and Respiratory Medicine, School of Biological Sciences, Faculty of Biology, Medicine and Health, The University of Manchester, Manchester, UK; 2 Royal Manchester Children's Hospital, Manchester University NHS Foundation Trust, Manchester, UK; 3 Lancaster University, Lancaster, UK; 4 Alder Hey Children's NHS Foundation Trust, Liverpool, UK; 5 Division of Pharmacy & Optometry, School of Health Sciences, Faculty of Biology, Medicine and Health, The University of Manchester, Manchester, UK; 6 NIHR Greater Manchester Patient Safety Research Collaboration (PSRC), The University of Manchester, Manchester, UK; 7 Centre for Academic Primary Care, Bristol Medical School: Population Health Sciences, University of Bristol, Bristol, UK

**Keywords:** paediatric otolaryngology, epidemiologic studies, qualitative research

## Abstract

**Introduction:**

Paediatric otorrhoea (PO) refers to the leakage of fluid through a perforation in the ear drum, resulting from an infection of the middle ear of a child or young person (CYP). PO frequently results in hearing loss which may lead to developmental delay, restricted communication and reduced educational attainment.

Epidemiological information for PO is largely derived from low-income countries. The aim of this study will be to establish the incidence of PO within the UK and to understand the impact of PO on CYP and their families’ everyday lives. It will build the foundations for a randomised controlled trial investigating the best antibiotic treatment for PO.

**Methods and analysis:**

The study will consist of two work packages. (1) Data from the Clinical Practice Research Datalink (CPRD), January 2005 to July 2021, will be used to determine the incidence of patient presentations with PO to primary care in the UK. It will also explore the current antimicrobial prescribing practice for PO in primary care. (2) Thirty semi-structured interviews will be conducted from 13 July to 31 October 2023 with CYP and their parents/carers to help identify the impact of PO on everyday life, the patient journey and how service users define treatment success. Three medical professional focus groups will be used to understand the current management practice, how treatment success is measured and acceptability to randomise patients. Thematic analysis will be used.

**Ethics and dissemination:**

The Health Research Authority, The Health and Social Care Research Ethics Committee (23/NI/0082) and the CPRD’s research data governance panel (22_002508) reviewed this study. Results will be disseminated at medical conferences, in peer-reviewed journals and via social media. The study will cocreate a webpage on healthtalk.org, with the Dipex Charity, about PO to ensure members of the public can learn more about the condition.

**Trial registration number:**

ISRCTN46071200.

**STRENGTHS AND LIMITATIONS OF THIS STUDY**

- Patient and public involvement has been used to shape the study aims and design.
- The Clinical Practice Research Datalink (CPRD) provides access to anonymised population-based electronic health records from general practices in the UK which will help determine the incidence and antibiotic treatment for paediatric otorrhoea.
- The limitation of CPRD database is that missing data is inevitable due to coding variation and incidence rates will be based solely on primary care presentations.
- Involvement of patients, their parents/carers and medical professionals will provide a range of experiences.
- The qualitative results will identify the most common themes and will not encompass all possible patient or medical professional experiences.

## Introduction

Paediatric otorrhoea (PO) is discharged from a child’s ear, that usually results from an infection of the middle ear. The accumulation of pus in the middle ear space causes the tympanic membrane to stretch until it ruptures, with the resultant leakage of foul-smelling discharge from the ear; called acute otitis media with discharge (AOMd). If AOMd persists it can lead to a permanent perforation of the ear drum with chronic discharge. The WHO classifies otorrhoea lasting more than 2 weeks as chronic suppurative otitis media (CSOM), although various alternative time frames are used in the medical literature.^1^

In routine clinical practice, classification systems for ear infections based on chronicity are not widely adopted by clinicians as they are not considered helpful in guiding management decisions. The presence, or absence, of otorrhoea is considered of greater relevance to treatment decisions. Our study group prefers to use the term PO to encompass the single disease process (AOMd and CSOM) as it translates more readily into clinical practice.

It is estimated that PO affects 50 million children and young people (CYP) per year globally.^2–4^ PO may cause significant temporary or permanent morbidity in children, most frequently resulting in hearing loss, which may then lead to developmental delay, restricted communication, poor psychosocial development and reduced educational attainment.^1^ The WHO estimates that chronically discharging ears account for over half of hearing disability globally.^1^ In addition, significant complications can occur, resulting in temporary or permanent sequelae such as imbalance, facial weakness and brain infections.

The psychosocial impact of PO on CYP and their care givers is poorly understood. Caregiver concern has been shown to be more prominent than the physical symptoms of PO in the limited published literature.^5^ Public involvement (CYP and their carers) during development of this study was supported by a National Institute for Health and Care Research (NIHR) Research Design Service (RDS) public involvement grant (RDSNW3687). Experts-by-experience described years of recurrent ‘smelly’ discharge negatively impacting on education, sporting activities and socialisation. CYP that were affected, as well as carers, explained that educators and family members seemed to not understand the condition. Caregivers felt that more effective treatment and quicker referral to secondary care was required.

The aim of this study is to develop high-quality data to form the foundations for a randomised controlled trial (RCT) investigating the management options for PO. This will be achieved through the following objectives:

- Determine the incidence and treatment for PO in the UK.
- Improve the understanding of the impact of PO on CYP and carers to ensure clinically meaningful outcome measures are selected.
- Understand how and why CYP with PO are currently treated and to assess treatment and randomisation acceptability for CYP, their carers and healthcare professionals for a future RCT.

## Methods and analysis

### Study design

**Figure 1:**
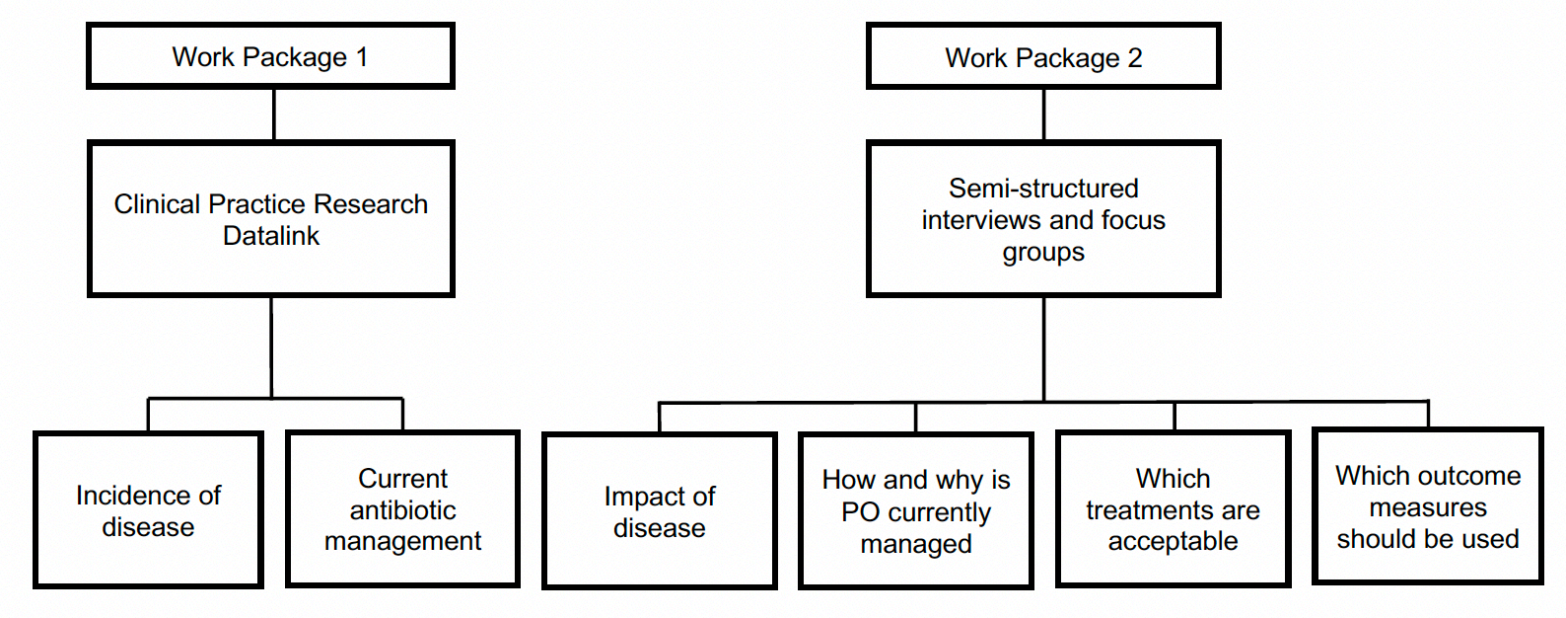
Study work packages and linked study objectives. PO, paediatric otorrhoea.

This study will be divided into two work packages to answer the research objectives (figure 1).

### Patient and public involvement

This study has codesign and coproduction running as a theme throughout. A (RDS) public involvement grant allowed the involvement of CYP with PO and their parents/carers during the study design stage. Their suggestions and experiences helped to shape every aspect of the study design. For example, parents/carers suggested either virtual meetings or phone calls were preferable to a face-to-face meeting due to their flexibility around childcare. This feedback is reflected in the study design.

A Patient Advisory Group (PAG) will be formed for the study, which will consist of 4–6 young people/parents/guardians who will meet at 3-month to 4-month intervals. The goals of the PAG are to help maintain the patient-focused nature of the work, maintain transparency, guide the design of the interview template and written patient information materials, as well as direct dissemination of the results.

To help determine the most effective methods of research dissemination and to help gain feedback on the design of a subsequent RCT, a study design workshop with public representatives will be conducted in January 2024. During the current study, we will identify and invite experts-by-experience to join the investigative team for the development of the subsequent research.

All members of the public will be supported by the patient and public involvement lead before, during and following the study. Training and support will be provided to all service users involved in this research.

### Work package 1

#### Design

Anonymised patient electronic health record data will be sourced from the CPRD. The CPRD contains electronic healthcare records from 1760 (21% of all) general practices, including over 16 million patients, in the UK.^6 7^ Of these patients, approximately 20% are aged under 18 years old.^8^ Data will be used from the Aurum dataset.

During the study period of interest, 1 January 2005 to 31 July 2021, CYP aged 16 years and below will be included. Patients will be followed up until: index PO episode, death, date of transfer out of practice, last date of data collection, study end date or their 16th birthday, whichever comes earliest.

We will calculate annual incidence rates for PO categorised by: biological sex assigned at birth, age, geographical location and index of multiple deprivation (IMD). We will generate these estimates by counting the number of cases and divide them by the total person-years at risk. The incidence will be presented as per 1000 person-years with its 95% CI. Antibiotic prescription data (by route and type) will also be examined.

#### Outcomes

Primary outcome measure: diagnosis with PO.

Secondary outcomes measures:

- Type and frequency of antibiotic prescriptions for PO.
- Frequency of referrals to secondary care ear, nose and throat services.

Factors measured:

- Biological sex at birth.
- Age.
- Geographical location.
- IMD.

#### Sample size

The proposed study is descriptive in design, therefore we do not have specific hypotheses and a formal sample size calculation is not required.

#### Limitations

The major limitations of the database are missing data and coding variation. Incomplete diagnoses, or those coded using free text, will not be identified by the CPRD. To ensure the correct patient presentations were identified an exhaustive list of codes related to PO was generated for the Aurum database (online supplemental file). Four clinicians reviewed the coding list. Codes were categorised as primary codes (correctly identify patients with otorrhoea), secondary codes (may identify patients with otorrhoea) or irrelevant coding. The secondary list will be used for a sensitivity analysis. Where clinicians disagreed with the coding decision, a consensus was reached following discussion. Codes were classified as secondary codes if there was no majority consensus to whether the code described PO. Codes relating to grommets or tympanostomy tubes were excluded as infection of grommets can also cause otorrhoea. Missing outcome data are not likely to be a major issue since diagnostic codes are binary and therefore indicate the presence or absence of clinical condition.

10.1136/bmjopen-2023-078052.supp1 Supplementary data

#### Analysis

Crude overall incidence rates (with 95% CIs) will be estimated. We will also produce internally age-standardised and sex-standardised incidence rates over the duration of the study period. The data will then be structured in an annual time-series format with event counts and ‘person-years at risk’ aggregated (by year) with stratification by sex, age group and deprivation (IMD) quintile. Mean-dispersion negative binomial regression models will be used to describe the trends in incidence rates over time. The natural logarithm of the denominator (person-years at risk) will be used as an offset in each regression model. To account for potential non-linear trends, calendar time will be modelled using splines or fractional polynomials. Practice-level variation will be examined using multi-level variants of the regression models with practice modelled using random effects. Sensitivity analyses will be undertaken to compare the incidence rates using the primary and secondary coding lists only.

### Work package 2

#### Study design

Semi-structured interviews will be conducted with CYP and their parents/carers, and focus groups with medical professionals. A semi-structured interview approach has been chosen due to its interactive and flexible nature, which is useful to capture the reality of everyday experience. The interview guide for the interviews and focus groups are expected to evolve following participant responses (online supplemental file). Inclusion criteria is outlined in table 1.

**Table 1:** Inclusion and exclusion criteria

Inclusion criteria	
Patient interviews	Children and young people aged 16 years and under. Children and young people who have had otorrhoea in the past year. Caregiver legally able to give consent for the child or young person (CYP) or the CYP themselves has the capacity to consent. Live in the UK. All languages (up to six non-English-speaking participants).
Medical professional focus groups	Medical professionals with experience of caring for CYP with otorrhoea in their clinical practice. Currently practicing in the UK. English-speaking.

#### Sample size

The recruitment aim for the patient interviews is 30 interviews or when data saturation is reached and the minimum number of patients with key demographics have participated (table 2). The recruitment aim for the medical professional focus groups is 24 participants divided over three focus groups. The sample size is estimated to allow for sufficient representation of the population of patients with PO. The study aims to capture a wide variety of experiences and practices within the medical professional cohort. Prior research studies addressing similar clinical questions and population were considered to reach the proposed sample size.^9^ It also takes into account the cost and time required to undertake 30 patient interviews and three focus groups.

**Table 2:** Minimum patient recruitment numbers by demographic

Demographic		Minimum number of Patients
Age	0–4 years	3
	5–8 years	3
	9–12 years	3
	13–16 years	3
Sex	Male	5
	Female	5
Disease presence	Active otorrhoea	4
	Previous otorrhoea within last year	4

### Recruitment

#### Patient interviews

Patient recruitment will use a direct-to-patient method by advertising the study using posters in medical establishments (eg, general practitioner (GP) practices and hospital outpatient waiting rooms), online (eg, charities, societies and institutions) and on social media.

The poster advertisement will direct the patient or carer to contact the research team by text, call or email. Recruitment will take place via a single institution. After the patient contacts the research team and expresses an interest in participating the cover letter, parent or carer information sheet and age-appropriate patient information sheet (PIS) will be sent to the participant.

The patient and parent/carer will be given the opportunity to read the study material to determine if they would like to participate. There will be a minimum of 2 days between sending the information material to the patient and arranging an interview.

In acknowledgement of participants’ valuable time, they will be remunerated with a gift £25 voucher in line with the NIHR guidelines for public involvement renumeration.^10^ Participants will be offered to be informed of the research findings.

#### Medical professional focus groups

Recruitment will use a direct-to-participant advertisement. A poster advertising the study will be sent for distribution through professional bodies, medical institutions and to primary and secondary care settings. The study poster will direct medical professionals to contact the research team to take part. Once they contact the research team, they will be sent the medical professional cover letter and PIS and booked onto a focus group session.

All participants will be offered to be informed of the research findings and will be acknowledged individually in publications produced from this work.

### Consent

Informed consent will be obtained prior to the participants undergoing any study related activities. The qualitative researcher will lead the consent discussion with all participants and will have up-to-date Good Clinical Practice training.

To improve dissemination of the research findings, the study team is working with the Dipex charity to create a webpage on https://healthtalk.org where information about PO can be shared publicly. The qualitative researcher will invite patients (CYP and their parents/carers) to be involved by contributing short clips of their video recording, audio recording or a transcript of their interview to the web page. Participating in this aspect of the study is optional and does not preclude the patients from taking part in the study.

### Data collection

#### Patient interviews

Interviews are expected to last between 45 and 60 min, but the length of the interview will be guided by the participant. The patient’s age at the time of interview and sex will be collected from the patient or parent/carer.

Parents/carers will be invited to take part in the interview on behalf of children younger than 5 years old. CYP who are 5 years or older will be invited to participate in joint interviews with their parents/carers, to recognise the importance of their experiences.

The study aims to reduce barriers to participation by using interpreters in the study. Sign language interpreters will be available for virtual or face-to-face interviews if required. An interpreter will be used for non-English-speaking participants. The interpreter will be used for patient consent and interview. Written material will be translated into any language for up to six participants if required. This number is based on the proportion of non-English speaking people in England and Wales. Translation will be performed depending on patient preference. Where possible, to improve the interview dynamic, the interpreter and the interviewer will be in the same location if a virtual interview is taking place. Interviews involving an interpreter will be allocated additional time to allow for translation time.

#### Medical professional focus groups

Semi-structured focus groups will be conducted with groups consisting of 6–8 participants. Nurses, audiologists, allied medical professionals, GPs, emergency department and otolaryngology doctors will be invited to participate. Healthcare professionals from varied demographic locations and of different grades will be invited to take part in the study. Focus groups will last for up to 60 min.

### Data analysis

Audio recording will be transcribed verbatim. The anonymised transcriptions will be imported to qualitative data analysis software for data management. Thematic analysis will follow guidance provided by Braun and Clarke who suggest the five key stages of thematic analysis include: (1) familiarising with the data; (2) generating codes; (3) identifying themes; (4) reviewing themes and collating into a thematic map or matrix; (5) write up the findings.^11^ We will also refer to Braun and Clarke’s recent assessment guidelines for evaluating thematic analysis research quality to ensure a well-developed and justified analysis, and to avoid the most common problems with this approach (‘proceduralism’ or a non-reflexive grouping of themes).^12^ There will be multiple independent coders drawn from the research team, as well as inter-rater reliability measures applied to the iterative process from codes to themes.^11 13^ The researchers will read and re-read the transcript to familiarise themselves with the data. Codes will then be generated in a systematic manner across the data set. Subsequently, the codes will be collated into potential themes which will then be reviewed and refined. Two researchers will independently identify themes before discussion in order to reach consensus.

## Ethics and dissemination

The CPRD protocol was approved by the CPRD’s research data governance process (22_002508) on 4 April 2023. The qualitative study was approved by the Health Research Authority on 23 June 2023 and the Research Ethics Committee (REC 23/NI/0082) on 15 June 2023.

This study will work with PAG members, service users involved in the study workshop and the Dipex charity to formulate an engagement plan to ensure the research findings are heard by the relevant audience. We will develop online public resources on healthtalk.org with the Dipex charity about PO. We aim to empower PAG members to help disseminate the research outcomes via local networks and online forums. Results will be disseminated to medical professionals at conferences, in peer-reviewed journals and via social media.

10.1136/bmjopen-2023-078052.supp2 Supplementary data

## Supplementary Material

Reviewer comments

Author's manuscript
