# Supplementary material for: Protocol for the Paediatric Otorrhoea Study (POSt): a multi-methods study to understand the burden of paediatric otorrhoea in the UK
Source: BMJ Open. 2023 Sep 5;13(9):e078052. doi: 10.1136/bmjopen-2023-078052 (PMC10481712; doi:10.1136/bmjopen-2023-078052)
Supplement: Supplementary data [file bmjopen-2023-078052supp001.pdf]

POSt Patient Interview Guide

Welcome and introduction - Seek verbal consent to continue, reminder of audio-recording of interview (or video recording if on Microsoft Teams), re-cap of project and plan for interview.

The study is aiming to understand what it is like for children and young people to live with an ear infection which causes a leaky ear. The interviews will explore your views and we are interested in your experience. It will help us to understand more about children and families' experiences of leaky ears. We also wanted to talk to you about how we might conduct research in the future on leaky ears, and if you think it is acceptable way of doing things. With this information we hope to help change how we treat children and young people with leaky ears.

If you do not feel you are able to comment on any area, please say so and we can stop at any time. Do you have any questions before we start?

1. Patient and carer experience of the condition

2. Patient and carer experience of treatment

3. Identifying in the patient and carer's opinion what is the best sign to use for treatment success

Lay explanation of randomised controlled trial is given by facilitator.

4. Identifying in the patient and carer's opinion on randomisation in a future trial

5. Identifying in the patient and carer's opinion on taking placebo medications

6. Identifying in the patient and carer's opinion on motivators and barriers to participating in a randomised controlled trial

Anything not covered?

Is there anything that we haven't covered in the interview that you think we should know or think about?

Closing and thanks - Thank for their time and contribution.

POSt Medical Professional Interview Guide

Welcome and introduction - Seek verbal consent to continue, and check that all members must interact with patients aged 16 and below with paediatric otorrhoea in daily practice to be eligible to take part. Provide a reminder of the video-recording of focus group (Microsoft Teams) will be saved, re-cap of project and plan for focus group discussion. Perform member check after each question or where necessary.

The study is aiming to understand how and why children and young people with PO are treated in primary and secondary care and to understand what treatments are acceptable to medical professionals. We are interested in your perceptions, based on your knowledge and experience. If you do not feel you are able to comment on any area, please say so. Do you have any questions before we start?

1. Introduction of group members and job role

2. Experiences of managing children and young people with paediatric otorrhoea

3. Normal treatment practices

4. Identifying treatment success

5. Motivations and barriers to participating in a future randomised controlled trial

Anything not covered?

Is there anything that we haven't covered in the focus group that you think we should know or think about?

Closing and thanks - Thank for their time and contribution.
