# Supplementary material for: Protocol for the Paediatric Otorrhoea Study (POSt): a multi-methods study to understand the burden of paediatric otorrhoea in the UK
Source: BMJ Open. 2023 Sep 5;13(9):e078052. doi: 10.1136/bmjopen-2023-078052 (PMC10481712; doi:10.1136/bmjopen-2023-078052)
Supplement: Supplementary data [file bmjopen-2023-078052supp002.pdf]

medcodeid	readcode	snomedctconceptid	snomedctdescriptionid	Aurum Database	Coding Selection
8.45868E+15		1.08256E+15	3078540019	Recurrent acute suppurative otitis media with spontaneous rupture of ear drum	Primary
253190015	1C4Z.00	162362000	253183013	Ear discharge symptom NOS	Primary
253183013	1C4..00	162362000	253183013	Ear discharge symptoms	Primary
4.5481E+15		162364004	253187014	Discharge from ear	Primary
4.54811E+15		162364004	253188016	Discharging ear	Primary
253186017	1C42.00	162364004	253186017	Ear discharge present	Primary
255616012	2D6..00	164211000	255616012	O/E - discharge from ear	Primary
255623013	2D6Z.00	164211000	255616012	O/E - ear discharge NOS	Primary
4.57677E+15		164211000	2667264011	On examination - discharge from ear	Primary
255618013	2D62.00	164213002	255618013	O/E - serous ear discharge	Primary
4.57681E+15		164213002	2667266013	On examination - serous ear discharge	Primary
255620011	2D64.00	164215009	255620011	O/E - purulent ear discharge	Primary
4.57685E+15		164215009	2666962013	On examination - purulent ear discharge	Primary
5.8754E+15		300132001	1490583015	Discharge from ear	Primary
5.87537E+15		300132001	1477433018	Ear discharge	Primary
5.87538E+15		300132001	441141019	Finding of ear discharge	Primary
5.87539E+15		300132001	1226738019	Observation of ear discharge	Primary
3.06032E+15		34790005	58064015	Benign chronic suppurative otitis media with anterior perforation of ear drum	Primary
3.11845E+15		38394007	490747016	CSOM - Chronic suppurative otitis media	Primary
299067013	F523.00	38394007	63479018	Chronic suppurative otitis media	Primary
299529015	FyuP200	38394007	63478014	[X]Other chronic suppurative otitis media	Primary
6.22921E+14	F586011	65668001	109100010	Discharge of ear	Primary
3.56778E+15		65668001	109101014	Drainage from external ear canal	Primary
3.56776E+15		65668001	109096014	Otorrhea	Primary
109102019	F586.00	65668001	109102019	Otorrhoea	Primary
8.83841E+14	F586.99	65668001	8.83841E+14	Otorrhoea - discharging ear	Primary

Heward E, *et al. BMJ Open* 2024; 13:e078052. doi: 10.1136/bmjopen-2023-078052

299229016	F586z00	65668001	109102019	Otorrhoea NOS	Primary
399506010	F586000	65668001	109102019	Unspecified otorrhoea	Primary
1.27288E+16		6.94131E+14	1.51841E+15	Chronic suppurative otitis media NOS	Primary
4.60551E+14	F520100	86279000	1235118013	Acute suppurative otitis media - tympanic membrane ruptured	Primary
3.90297E+15		86279000	1235119017	Acute suppurative otitis media with discharge	Primary
3.90295E+15		86279000	143085014	Acute suppurative otitis media with spontaneous rupture of ear drum	Primary
5.56821E+14	F521.00	87665008	1235262012	Chronic suppurative otitis media - tubotympanic	Primary
3.92483E+15		87665008	145352014	Chronic tubotympanic suppurative otitis media	Primary
4.06248E+15		95803004	158677012	Serous drainage from external ear canal	Primary
4.06249E+15		95804005	158678019	Purulent drainage from external ear canal	Primary
4.06251E+15		95805006	158679010	Foul odor drainage from external ear canal	Primary
4.0625E+15		95805006	201783016	Foul odour drainage from external ear canal	Primary
3.11848E+15		38394007	490751019	Chronic otitis media with perforation	Secondary
3.17615E+15		41954005	70007011	Chronic atticoantral suppurative otitis media	Secondary
5.56811E+14	F522.00	41954005	1229812011	Chronic suppurative otitis media - atticoantral	Secondary
4.77469E+15		194281003	299062019	ASOM - Acute suppurative otitis media	Secondary
4.7747E+15		194281003	299064018	Acute purulent otitis media	Secondary
299061014	F520.00	194281003	299061014	Acute suppurative otitis media	Secondary
299066016	F520z00	194281003	299061014	Acute suppurative otitis media NOS	Secondary
4.77471E+15		194282005	2575709017	Acute suppurative otitis media due to another disease	Secondary
299065017	F520300	194282005	299065017	Acute suppurative otitis media due to disease EC	Secondary
299069011	F524000	194286008	299069011	Bilateral suppurative otitis media	Secondary
3.06031E+15		34790005	58063014	Chronic tubotympanic disease with anterior perforation of ear drum	Secondary
490746013	F513100	38394007	490746013	Chronic otitis media with effusion, purulent	Secondary
8.83741E+14	F523.99	6.94131E+14	8.83741E+14	Chronic purulent otitis media	Secondary
490749018	F513111	38394007	490749018	Chronic secretory otitis media, purulent	Secondary
3.11847E+15		38394007	490750018	Otitis media with effusion - purulent	Secondary

Heward E, et al. BMJ Open 2024; 13:e078052. doi: 10.1136/bmjopen-2023-078052

1.16801E+14	F52..00	39288006	65880011	Purulent otitis media	Secondary
299068015	F524.00	39288006	65880011	Purulent otitis media NOS	Secondary
3.13204E+15		39288006	65883013	Suppurative otitis media	Secondary
3.17617E+15		41954005	70011017	Persistent mucosal disease with posterior AND/OR superior marginal perforation of ear drum	Secondary
8.83741E+14	F523.99	6.94131E+14	8.83741E+14	Chronic purulent otitis media	Secondary
7.98739E+15		7.361E+12	3022971014	Perforation of tympanic membrane due to otitis media	Secondary
504727012	F511.11	81564005	504727012	Chronic secretory otitis media, serous	Secondary
135302019	F511.99	81564005	8.83691E+14	Chronic serous otitis media	Secondary
299024017	F511z00	81564005	135302019	Chronic serous otitis media	Secondary
3.82626E+15		81564005	504725016	Glue ear - serous	Secondary
5.55791E+14	F511.00	81564005	504726015	Otitis media with effusion - serous	Secondary
3.82625E+15		81564005	135305017	Simple chronic serous otitis media	Secondary
299023011	F511300	81564005	135302019	Unilateral chronic serous otitis	Secondary
5.0338E+15		232251007	347975014	Recurrent acute suppurative otitis media	Secondary
6.01386E+15		312218008	455892014	Infective otitis media	Secondary
11759018	F512100	6485001	11759018	Mucosanguinous chronic otitis media	Secondary
7.57612E+15		703469002	3008656010	Bacterial otitis media	Secondary
7.82221E+15		721742004	3325908013	Otitis media caused by Streptococcus pneumoniae	Secondary
7.82222E+15		721742004	3325909017	Streptococcus pneumoniae otitis media	Secondary

Heward E, *et al. BMJ Open* 2024; 13:e078052. doi: 10.1136/bmjopen-2023-078052
