# Supplementary material for: Protocol for the Paediatric Otorrhoea Study (POSt): a multi-methods study to understand the burden of paediatric otorrhoea in the UK
Source: BMJ Open. 2023 Sep 5;13(9):e078052. doi: 10.1136/bmjopen-2023-078052 (PMC10481712; doi:10.1136/bmjopen-2023-078052)
Supplement: Author's manuscript [file bmjopen-2023-078052.draft_revisions.pdf]

BMJ Open is committed to open peer review. As part of this commitment we make the peer review history of every article we publish publicly available.

When an article is published we post the peer reviewers' comments and the authors' responses online. We also post the versions of the paper that were used during peer review. These are the versions that the peer review comments apply to.

The versions of the paper that follow are the versions that were submitted during the peer review process. They are not the versions of record or the final published versions. They should not be cited or distributed as the published version of this manuscript.

BMJ Open is an open access journal and the full, final, typeset and author-corrected version of record of the manuscript is available on our site with no access controls, subscription charges or pay-per-view fees (http://bmjopen.bmj.com).

If you have any questions on BMJ Open's open peer review process please email info.bmjopen@bmj.com

BMJ Open

The Paediatric Otorrhoea Study (POSt): Understanding the burden of disease and acceptability of non-surgical treatment options

Journal:	*BMJ Open*
Manuscript ID	bmjopen-2023-078052
Article Type:	Protocol
Date Submitted by the Author:	22-Jul-2023

Complete List of Authors:	Heward, Elliot; The University of Manchester, Division of Infection, Immunity and Respiratory Medicine, School of Biological Sciences, Faculty of Biology, Medicine and Health<br>Dempsey, James; Royal Manchester Children's Hospital<br>Lunn, Judith; Lancaster University<br>Molloy, John; Royal Manchester Children's Hospital<br>Isba, Rachel; Lancaster University<br>Carr, M; The University of Manchester, Division of Pharmacy & Optometry, School of Health Sciences, Faculty of Biology, Medicine and Health<br>Ashcroft, Darren; The University of Manchester, Division of Pharmacy & Optometry, School of Health Sciences, Faculty of Biology, Medicine and Health<br>Hay, Alastair; University of Bristol, Centre for Academic Primary Care, Bristol Medical School: Population Health Sciences<br>Nichani, Jaya; Royal Manchester Children's Hospital<br>Bruce, Iain; Royal Manchester Children's Hospital

Keywords:	OTOLARYNGOLOGY, Paediatric otolaryngology < OTOLARYNGOLOGY, EPIDEMIOLOGIC STUDIES, QUALITATIVE RESEARCH

SCHOLARONE™
Manuscripts

Abstract

Introduction

Paediatric otorrhoea (PO) refers to the leakage of fluid through a perforation in the ear drum, resulting from an infection of the middle ear of a child or young person (CYP). PO frequently results in hearing loss which may lead to developmental delay, restricted communication and reduced educational attainment.

Epidemiological information for PO is largely derived from low-income countries. The aim of this study will be to establish the incidence of PO within the UK and to understand the impact of PO on CYP and their families' everyday lives. It will build the foundations for a randomised controlled trial investigating the best antibiotic treatment for PO.

Methods and Analysis

The study will consist of two work packages. 1) Data from the Clinical Practice Research Datalink (CPRD), January 2005 to July 2021, will be used to determine the incidence of patient presentations with PO to primary care in the UK. It will also explore the current antimicrobial prescribing practice for PO in primary care. 2) Thirty semi-structured interviews will be conducted with CYP and their parents/carers to help identify the impact of PO on everyday life, the patient journey and how service users define treatment success. Three medical professional focus groups will be used to understand the current management practice, how treatment success is measured and acceptability to randomise patients. Thematic analysis will be used.

Ethics and dissemination

The Health Research Authority, The Health and Social Care Research Ethics Committee (23/NI/0082) and the CPRD's research data governance panel (22_002508) reviewed this study. Results will be disseminated at medical conferences, in peer-reviewed journals, via social media and on the sponsor's webpage. The study will co-create a webpage on healthtalk.org, with the Dipex Charity, about PO so to ensure members of the public can learn more about the condition.

For peer review only

Strengths and Limitations of the Study

- Patient and public involvement has been used to shape the study aims and design.
- The CPRD provides access to anonymised population-based electronic health records from general practices in the UK which will help determine the incidence and antibiotic treatment for paediatric otorrhoea.
- The CPRD database does not include information on medicines prescribed from hospitals.
- Involvement of patients, their parents/carers and medical professionals will provide a range of experiences.
- The qualitative results will identify the most common themes and will not encompass all possible patient or medical professional experiences.

Introduction

Paediatric otorrhoea (PO) is discharge from a child's ear, that usually results from an infection of the middle ear. The accumulation of pus in the middle ear space causes the tympanic membrane to stretch until it ruptures, with the resultant leakage of foul-smelling discharge from the ear; called Acute Otitis Media with discharge (AOMd). If AOMd persists it can lead to a permanent perforation of the ear drum with chronic discharge. The World Health Organization (WHO) classifies otorrhoea lasting more than two weeks as Chronic Suppurative Otitis Media (CSOM), although various alternative time frames are used in the medical literature [1].

In routine clinical practice, classification systems for ear infections based on chronicity are not widely adopted by clinicians as they are not considered helpful in guiding management decisions. The presence, or absence, of otorrhoea being considered of greater relevance to treatment decisions. Our study group prefers to use the term Paediatric Otorrhoea (PO) to encompass the single disease process (AOMd and CSOM) as it translates more readily into clinical practice.

It is estimated that PO affects 50 million CYP per year globally [2-4]. PO may cause significant temporary or permanent morbidity in children, most frequently resulting in hearing loss, which may then lead to developmental delay, restricted communication, poor psychosocial development, and reduced educational attainment[1]. The WHO estimates that chronically discharging ears account for over half of hearing disability globally[1]. In addition, significant complications can occur, resulting in temporary or permanent sequelae such as imbalance, facial weakness and brain infections.

For peer review only - http://bmjopen.bmj.com/site/about/guidelines.xhtml

The psychosocial impact of PO on children and young people (CYP) and their care givers is poorly understood. Caregiver concern has been shown to be more prominent than the physical symptoms of PO in the limited published literature [5]. Public involvement (CYP and their carers) during development of this study was supported by a National Institute for Health and Care Research (NIHR) Research Design Service (RDS) public involvement grant (RDSNW3687). Experts-by-experience described years of recurrent ‘smelly ’discharge negatively impacting on education, sporting activities and socialisation. CYP that were affected, as well as carers, explained that educators and family members seemed to not understand the condition. Caregivers felt that more effective treatment and quicker referral to secondary care was required.

The aim of this study is to develop high-quality data to form the foundations for a randomised controlled trial (RCT) investigating the management options for PO. This will be achieved through the following objectives:

- determine the incidence and treatment for PO in the UK;
- improve the understanding of the impact of PO on CYP and carers to ensure clinically meaningful outcome measures are selected;
- understand how and why CYP with PO are currently treated and to assess treatment and randomisation acceptability for CYP, their carers and healthcare professionals for a future RCT.

Methods and Analysis

Study Design

This study will be divided into two work packages to answer the research objectives (Figure 1).

Patient and Public Involvement

This study has co-design and co-production running as a theme throughout. A (RDS) public involvement grant allowed the involvement of CYP with PO and their parents/carers during the study design stage. Their suggestions and experiences helped to shape every aspect of the study design. For example, parents/carers suggested either virtual meetings or phone calls were preferable to a face-to-face meeting due to their flexibility around childcare. This feedback is reflected in the study design.

A Patient Advisory Group (PAG) will be formed for the study, which will consist of 4-6 young people/parents/guardians who will meet at 3-to-4-monthly intervals. The goals

For peer review only - http://bmjopen.bmj.com/site/about/guidelines.xhtml

of the PAG are to help maintain the patient-focused nature of the work, maintain transparency, guide the design of the interview template and written patient information materials, as well as direct dissemination of the results.

To help determine the most effective methods of research dissemination and to help gain feedback on the design of a subsequent RCT, a study design workshop with public representatives will be conducted in January 2024. During the current study, we will identify and invite experts-by-experience to join the investigative team for the development of the subsequent research.

All members of the public will be supported by the PPI lead before, during and following the study. Training and support will be provided to all service users involved in this research.

Work Package 1

Design

Anonymised patient electronic health record data will be sourced from the CPRD. The CPRD contains electronic healthcare records from 1,760 (21% of all) general practices, including over 16 million patients, in the UK [6,7]. Of these patients, approximately 20% are aged under 18 years old [8]. Data will be utilised from the Aurum dataset.

During the study period of interest, 1st January 2005 to 31st July 2021, CYP aged 16 years and below will be included. Patients will be followed up until: index PO episode, death, date of transfer out of practice, last date of data collection, study end date or their 16th birthday, whichever comes earliest.

We will calculate annual incidence rates for PO categorised by: biological sex assigned at birth, age, geographical location and Index of Multiple Deprivation (IMD). We will generate these estimates by counting the number of cases and divide them by the total person-years at risk. The incidence will be presented as per 1000 person-years with its 95% confidence interval. Antibiotic prescription data (by route and type) will also be examined.

Outcomes

Primary outcome measure: diagnosis with paediatric otorrhoea

Secondary outcomes measures:

- type and frequency of antibiotic prescriptions for PO;
- frequency of referrals to secondary care ear, nose and throat services.

For peer review only - http://bmjopen.bmj.com/site/about/guidelines.xhtml

Factors measured:

- biological Sex at Birth
- age
- geographical location
- index of Multiple Deprivation

Sample Size

The proposed study is descriptive in design, therefore we do not have specific hypotheses and a formal sample size calculation is not required.

Limitations

The major limitations of the database are missing data and coding variation. Incomplete diagnoses, or those coded using free text, will not be identified by the CPRD. To ensure the correct patient presentations were identified an exhaustive list of codes related to PO was generated for the Aurum database (Appendix. CPRD Coding List). Four clinicians reviewed the coding list. Codes were categorised as primary codes (correctly identify patients with otorrhoea), secondary codes (may identify patients with otorrhoea) or irrelevant coding. The secondary list will be used for a sensitivity analysis. Where clinicians disagreed in the coding decision, a consensus was reached following discussion. Codes were classified as secondary codes if there was no majority consensus to whether the code described PO. Codes relating to grommets or tympanostomy tubes were excluded as infection of grommets can also cause otorrhoea. Missing outcome data are not likely to be a major issue since diagnostic codes are binary and therefore indicate the presence or absence of clinical condition.

Analysis

Crude overall incidence rates (with 95% confidence intervals) will be estimated. We will also produce internally age- and sex-standardised incidence rates over the duration of the study period. The data will then be structured in an annual time-series format with event counts and ‘person-years at risk’ aggregated (by year) with stratification by sex, age group and deprivation (IMD) quintile. Mean-dispersion negative binomial regression models will be used to describe the trends in incidence rates over time. The natural logarithm of the denominator (person-years at risk) will be used as an offset in each regression model. To account for potential non-linear trends, calendar time will be modelled using splines or fractional polynomials. Practice-level variation will be examined using multi-level variants of the regression models with practice modelled using random effects. Sensitivity analyses will be

For peer review only - http://bmjopen.bmj.com/site/about/guidelines.xhtml

undertaken to compare the incidence rates using the primary and secondary coding lists only.

Work Package 2

Study Design

Semi-structured interviews will be conducted with CYP and their parents/carers, and focus groups with medical professionals. A semi-structured interview approach has been chosen due to its interactive and flexible nature, which is useful to capture the reality of everyday experience. The interview guide for the interviews and focus groups are expected to evolve following participant responses. Inclusion criteria is outlined in Table 1.

Sample Size

The recruitment aim for the patient interviews is 30 interviews or when data saturation is reached and the minimum number of patients with key demographics have participated (Table 2). The recruitment aim for the medical professional focus groups is 24 participants divided over three focus groups. The sample size is estimated to allow for sufficient representation of the population of patients with PO. The study aims to capture a wide variety of experiences and practices within the medical professional cohort. Prior research studies addressing similar clinical questions and population were considered to reach the proposed sample size [9]. It also takes into account the cost and time required to undertake 30 patient interviews and three focus groups.

Recruitment

Patient Interviews

Patient recruitment will use a direct-to-patient method by advertising the study using posters in medical establishments (e.g. GP practices and hospital outpatient waiting rooms), online (e.g. charities, societies and institutions) and on social media.

The poster advertisement will direct the patient or carer to contact the research team by text, call or email. Recruitment will take place via a single institution. After the patient contacts the research team and expresses an interest in participating the cover letter, parent or carer information sheet and age-appropriate patient information sheet (PIS) will be sent to the participant.

For peer review only - http://bmjopen.bmj.com/site/about/guidelines.xhtml

The patient and parent/carer will be given the opportunity to read the study material to determine if they would like to participate. There will be a minimum of two days between sending the information material to the patient and arranging an interview.

In acknowledgement of participants' valuable time, they will be remunerated with a gift £25 voucher in line with the NIHR guidelines for public involvement renumeration [10]. Participants will be offered to be informed of the research findings.

Medical Professional Focus Groups

Recruitment will use a direct-to-participant advertisement. A poster advertising the study will be sent for distribution through professional bodies, medical institutions and to primary and secondary care settings. The study poster will direct medical professionals to contact the research team to take part. Once they contact the research team, they will be sent the medical professional cover letter and PIS and booked onto a focus group session.

All participants will be offered to be informed of the research findings and will be acknowledged individually in publications produced from this work.

Consent

Informed consent will be obtained prior to the participants undergoing any study related activities. The qualitative researcher will lead the consent discussion with all participants and will have up-to-date GCP training.

To improve dissemination of the research findings, the study team is working with the Dipex charity to create a webpage on https://healthtalk.org where information about PO can be shared publicly. The qualitative researcher will invite patients (CYP and their parents/carers) to be involved by contributing short clips of their video recording, audio recording or a transcript of their interview to the web page. Participating in this aspect of the study is optional and does not preclude the patients from taking part in the study.

Data Collection

Patient interviews

Interviews are expected to last between 45-60 minutes, but the length of the interview will be guided by the participant. The patient's age at the time of interview and sex will be collected from the patient or parent/carer.

For peer review only - http://bmjopen.bmj.com/site/about/guidelines.xhtml

Parents/carers will be invited to take part in the interview on behalf of children younger than 5 years old. CYP who are 5 years or older will be invited to participate in joint interviews with their parents/carers, to recognise the importance of their experiences.

The study aims to reduce barriers to participation by using interpreters in the study. Sign language interpreters will be available for virtual or face-to-face interviews if required. An interpreter will be used for non-English-speaking participants. The interpreter will be used for patient consent and interview. Written material will be translated into any language for up to 6 participants if required. This number is based on the proportion of non-English speaking people in England and Wales. Translation will be performed depending on patient preference. Where possible, to improve the interview dynamic, the interpreter and the interviewer will be in the same location if a virtual interview is taking place. Interviews involving an interpreter will be allocated additional time to allow for translation time.

Medical Professional Focus Groups

Semi-structured focus groups will be conducted with groups consisting of 6-8 participants. Nurses, audiologists, allied medical professionals, GPs, Emergency Department and Otolaryngology doctors will be invited to participate. Healthcare professionals from varied demographic locations and of different grades will be invited to take part in the study. Focus groups will last for up to 60 minutes.

Data Analysis

Audio recording will be transcribed verbatim. The anonymised transcriptions will be imported to qualitative data analysis software for data management. Thematic analysis will follow guidance provided by Braun and Clarke (2006) who suggest the five key stages of thematic analysis include: (1) familiarizing with the data (2) generating codes (3) identifying themes (4) reviewing themes and collating into a thematic map or matrix (5) write up the findings [11]. We will also refer to Braun and Clarke’s (2021) recent assessment guidelines for evaluating thematic analysis research quality to ensure a well-developed and justified analysis, and to avoid the most common problems with this approach (‘proceduralism’ or a non-reflexive grouping of themes) [12]. There will be multiple independent coders drawn from the research team, as well as inter-rater reliability measures applied to the iterative process from codes to themes [11,13]. The researchers will read and re-read the transcript to familiarise themselves with the data. Codes will then be generated in a systematic manner across the data set. Subsequently, the codes will be collated into potential themes which will then be reviewed and refined. Two researchers will independently identify themes before discussion in order to reach consensus.

For peer review only - http://bmjopen.bmj.com/site/about/guidelines.xhtml

Ethics and Dissemination

The Clinical Practice Research Datalink (CRPD) protocol was approved by the CPRD's research data governance process (*22_002508*) on 4 April 2023. The qualitative study was approved by The Health Research Authority on 23 June 2023 and The Research Ethics Committee (REC 23/NI/0082) on 15 June 2023.

This study will work with PAG members, service users involved in the study workshop and the Dipex charity to formulate an engagement plan to ensure the research findings are heard by the relevant audience. We will develop online public resources on healthtalk.org with the Dipex charity about PO. We aim to empower PAG members to help disseminate the research outcomes via local networks and online forums. Results will be disseminated to medical professionals at conferences, in peer-reviewed journals and via social media.

References

1. World Health Organization. Chronic suppurative otitis media. Burden of illness and management options. Available: https://apps.who.int/iris/handle/10665/42941 [Accessed 22 July 2023]
2. Little P, Gould C, Williamson I, *et al*. Pragmatic randomised controlled trial of two prescribing strategies for childhood acute otitis media. BMJ 2001;10:336-42. doi: 10.1136/bmj.322.7282.336
3. Monasta L, Ronfani L, Marchetti F, *et al*. Burden of disease caused by otitis media: systematic review and global estimates. PLoS One. 2012;7:e36226. doi: 10.1371/journal.pone.0036226
4. Amnesty International. Children's Human Rights. Available: https://www.amnesty.org/en/what-we-do/child-rights/ [Accessed 22 July 2023]
5. Vlastos IM, Kandiloros D, Manolopoulos L, *et el*. Quality of life in children with chronic suppurative otitis media with or without cholesteatoma. Int J Pediatr Otorhinolaryngol. 2009;73:363-9. doi: 10.1016/j.ijporl.2008.10.030
6. Clinical Practice Research Datalink. (2022). CPRD Aurum March 2022 (Version 2022.03.001) [Data set]. Clinical Practice Research Datalink. https://doi.org/10.48329/MY9S-4X08
7. Clinical Practice Research Datalink. (2022). CPRD GOLD March 2022 (Version 2022.03.001) [Data set]. Clinical Practice Research Datalink. https://doi.org/10.48329/5RHX-ER29

For peer review only - http://bmjopen.bmj.com/site/about/guidelines.xhtml

8. Wolf A, Dedman D, Campbell J, *et al*. Data resource profile: Clinical Practice Research Datalink (CPRD) Aurum. Int J Epidemiol. 2019;48:1740-1740g. doi: 10.1093/ije/dyz034

9. Vasileiou K, Barnett J, Thorpe S, *et al*. Characterising and justifying sample size sufficiency in interview-based studies: systematic analysis of qualitative health research over a 15-year period. BMC Med Res Methodol. 2018;18:148. doi: 10.1186/s12874-018-0594-7

10. NIHR. Payment guidance for researchers and professionals. Available: https://www.nihr.ac.uk/documents/payment-guidance-for-researchers-and-professionals/27392 [Accessed 22 July 2023]

11. Braun V, Clarke V. Using thematic analysis in psychology. Qualitative research in psychology. 2006;3:77-101. Doi: 10.1191/1478088706qp063oa

12. Braun V, Clarke V. Thematic Analysis: A Practical Guide. Sage publications, 2021

13. Braun V, Clarke V. One size fits all? What counts as quality practice in (reflexive) thematic analysis?. Qualitative research in psychology. 2021;18:328-52. Doi: 10.1080/14780887.2020.1769238

Authors Contributions

Study conception and overall study design (EH & IAB). Design of the public engagement elements (JD & JN) Design of work package 1 (MC, DA & AH). Design of work package 2 (JL, RI & JM). EH and JD drafted the manuscript which was reviewed by all authors.

Funding statement

This work was supported by the National Institute of Health and Care Research (NIHR) grant number NIHR204036. DMA is supported by the NIHR Greater Manchester Patient Safety Research Collaboration (PSRC).

Competing Interests

No completing interests declared.

Word Count: 2451

For peer review only - http://bmjopen.bmj.com/site/about/guidelines.xhtml

Tables

Table 1. Inclusion and Exclusion Criteria

**Inclusion Criteria**	
Patient Interviews	• Children and young people aged 16 years and under<br>• Children and young people who have had otorrhoea in the past year<br>• Caregiver legally able to give consent for the CYP or the CYP themselves has the capacity to consent<br>• Live in the United Kingdom (UK)<br>• All languages (up to six non-English-speaking participants)
Medical Professional Focus Groups	• Medical professionals with experience of caring for CYP with otorrhoea in their clinical practice<br>• Currently practicing in the UK<br>• English-speaking

Table 2. Minimum Patient Recruitment Numbers by Demographic

**Demographic**		**Minimum Number of Patients**
Age	0-4 years	3
	5-8 years	3
	9-12 years	3
	13-16 years	3
Sex	Male	5
	Female	5
Disease Presence	Active otorrhoea	4
	Previous otorrhoea within last year	4

For peer review only

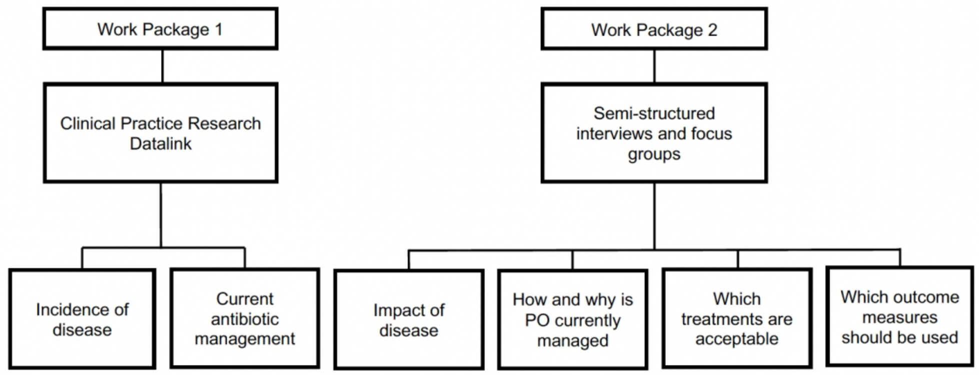

Figure 1. Study work packages and linked study objectives (PO = paediatric otorrhoea).

280x110mm (144 x 144 DPI)

medcodeid	readcode	snomedctconceptid	snomedctdescriptionid	Aurum Database	Coding Selection
8.45868E+15		1.08256E+15	3078540019	Recurrent acute suppurative otitis media with spontaneous rupture of ear drum	Primary
253190015	1C4Z.00	162362000	253183013	Ear discharge symptom NOS	Primary
253183013	1C4..00	162362000	253183013	Ear discharge symptoms	Primary
4.5481E+15		162364004	253187014	Discharge from ear	Primary
4.54811E+15		162364004	253188016	Discharging ear	Primary
253186017	1C42.00	162364004	253186017	Ear discharge present	Primary
255616012	2D6..00	164211000	255616012	O/E - discharge from ear	Primary
255623013	2D6Z.00	164211000	255616012	O/E - ear discharge NOS	Primary
4.57677E+15		164211000	2667264011	On examination - discharge from ear	Primary
255618013	2D62.00	164213002	255618013	O/E - serous ear discharge	Primary
4.57681E+15		164213002	2667266013	On examination - serous ear discharge	Primary
255620011	2D64.00	164215009	255620011	O/E - purulent ear discharge	Primary
4.57685E+15		164215009	2666962013	On examination - purulent ear discharge	Primary
5.8754E+15		300132001	1490583015	Discharge from ear	Primary
5.87537E+15		300132001	1477433018	Ear discharge	Primary
5.87538E+15		300132001	441141019	Finding of ear discharge	Primary
5.87539E+15		300132001	1226738019	Observation of ear discharge	Primary
3.06032E+15		34790005	58064015	Benign chronic suppurative otitis media with anterior perforation of ear drum	Primary
3.11845E+15		38394007	490747016	CSOM - Chronic suppurative otitis media	Primary
299067013	F523.00	38394007	63479018	Chronic suppurative otitis media	Primary
299529015	FyuP200	38394007	63478014	[X]Other chronic suppurative otitis media	Primary
6.22921E+14	F586011	65668001	109100010	Discharge of ear	Primary
3.56778E+15		65668001	109101014	Drainage from external ear canal	Primary
3.56776E+15		65668001	109096014	Otorrhea	Primary
109102019	F586.00	65668001	109102019	Otorrhoea	Primary
8.83841E+14	F586.99	65668001	8.83841E+14	Otorrhoea - discharging ear	Primary

For peer review only

299229016	F586z00	65668001	109102019	Otorrhoea NOS	Primary
399506010	F586000	65668001	109102019	Unspecified otorrhoea	Primary
1.27288E+16		6.94131E+14	1.51841E+15	Chronic suppurative otitis media NOS	Primary
4.60551E+14	F520100	86279000	1235118013	Acute suppurative otitis media - tympanic membrane ruptured	Primary
3.90297E+15		86279000	1235119017	Acute suppurative otitis media with discharge	Primary
3.90295E+15		86279000	143085014	Acute suppurative otitis media with spontaneous rupture of ear drum	Primary
5.56821E+14	F521.00	87665008	1235262012	Chronic suppurative otitis media - tubotympanic	Primary
3.92483E+15		87665008	145352014	Chronic tubotympanic suppurative otitis media	Primary
4.06248E+15		95803004	158677012	Serous drainage from external ear canal	Primary
4.06249E+15		95804005	158678019	Purulent drainage from external ear canal	Primary
4.06251E+15		95805006	158679010	Foul odor drainage from external ear canal	Primary
4.0625E+15		95805006	201783016	Foul odour drainage from external ear canal	Primary
3.11848E+15		38394007	490751019	Chronic otitis media with perforation	Secondary
3.17615E+15		41954005	70007011	Chronic atticoantral suppurative otitis media	Secondary
5.56811E+14	F522.00	41954005	1229812011	Chronic suppurative otitis media - atticoantral	Secondary
4.77469E+15		194281003	299062019	ASOM - Acute suppurative otitis media	Secondary
4.7747E+15		194281003	299064018	Acute purulent otitis media	Secondary
299061014	F520.00	194281003	299061014	Acute suppurative otitis media	Secondary
299066016	F520z00	194281003	299061014	Acute suppurative otitis media NOS	Secondary
4.77471E+15		194282005	2575709017	Acute suppurative otitis media due to another disease	Secondary
299065017	F520300	194282005	299065017	Acute suppurative otitis media due to disease EC	Secondary
299069011	F524000	194286008	299069011	Bilateral suppurative otitis media	Secondary
3.06031E+15		34790005	58063014	Chronic tubotympanic disease with anterior perforation of ear drum	Secondary
490746013	F513100	38394007	490746013	Chronic otitis media with effusion, purulent	Secondary
8.83741E+14	F523.99	6.94131E+14	8.83741E+14	Chronic purulent otitis media	Secondary
490749018	F513111	38394007	490749018	Chronic secretory otitis media, purulent	Secondary
3.11847E+15		38394007	490750018	Otitis media with effusion - purulent	Secondary

For peer review only

1.16801E+14	F52..00	39288006	65880011	Purulent otitis media	Secondary
299068015	F524.00	39288006	65880011	Purulent otitis media NOS	Secondary
3.13204E+15		39288006	65883013	Suppurative otitis media	Secondary
3.17617E+15		41954005	70011017	Persistent mucosal disease with posterior AND/OR superior marginal perforation of ear drum	Secondary
8.83741E+14	F523.99	6.94131E+14	8.83741E+14	Chronic purulent otitis media	Secondary
7.98739E+15		7.361E+12	3022971014	Perforation of tympanic membrane due to otitis media	Secondary
504727012	F511.11	81564005	504727012	Chronic secretory otitis media, serous	Secondary
135302019	F511.99	81564005	8.83691E+14	Chronic serous otitis media	Secondary
299024017	F511z00	81564005	135302019	Chronic serous otitis media	Secondary
3.82626E+15		81564005	504725016	Glue ear - serous	Secondary
5.55791E+14	F511.00	81564005	504726015	Otitis media with effusion - serous	Secondary
3.82625E+15		81564005	135305017	Simple chronic serous otitis media	Secondary
299023011	F511300	81564005	135302019	Unilateral chronic serous otitis	Secondary
5.0338E+15		232251007	347975014	Recurrent acute suppurative otitis media	Secondary
6.01386E+15		312218008	455892014	Infective otitis media	Secondary
11759018	F512100	6485001	11759018	Mucosanguinous chronic otitis media	Secondary
7.57612E+15		703469002	3008656010	Bacterial otitis media	Secondary
7.82221E+15		721742004	3325908013	Otitis media caused by Streptococcus pneumoniae	Secondary
7.82222E+15		721742004	3325909017	Streptococcus pneumoniae otitis media	Secondary

For peer review only

For peer review only - http://bmjopen.bmj.com/site/about/guidelines.xhtml

BMJ Open

Protocol for the Paediatric Otorrhoea Study (POSt): a multi-methods study to understand the burden of paediatric otorrhoea in the UK

Journal:	*BMJ Open*
Manuscript ID	bmjopen-2023-078052.R1
Article Type:	Protocol
Date Submitted by the Author:	27-Jul-2023
Complete List of Authors:	Heward, Elliot; The University of Manchester, Division of Infection, Immunity and Respiratory Medicine, School of Biological Sciences, Faculty of Biology, Medicine and Health<br>Dempsey, James; Royal Manchester Children's Hospital<br>Lunn, Judith; Lancaster University<br>Molloy, John; Royal Manchester Children's Hospital; The University of Manchester, Division of Infection, Immunity and Respiratory Medicine, School of Biological Sciences, Faculty of Biology, Medicine and Health<br>Isba, Rachel; Lancaster University; Alder Hey Children's NHS Foundation Trust<br>Carr, Matthew; The University of Manchester, Division of Pharmacy & Optometry, School of Health Sciences, Faculty of Biology, Medicine and Health<br>Ashcroft, Darren; The University of Manchester, Division of Pharmacy & Optometry, School of Health Sciences, Faculty of Biology, Medicine and Health; The University of Manchester, NIHR Greater Manchester Patient Safety Research Collaboration (PSRC)<br>Hay, Alastair; University of Bristol, Centre for Academic Primary Care, Bristol Medical School: Population Health Sciences<br>Nichani, Jaya; Royal Manchester Children's Hospital; The University of Manchester, Division of Infection, Immunity and Respiratory Medicine, School of Biological Sciences, Faculty of Biology, Medicine and Health<br>Bruce, Iain; Royal Manchester Children's Hospital; The University of Manchester, Division of Infection, Immunity and Respiratory Medicine, School of Biological Sciences, Faculty of Biology, Medicine and Health
**Primary Subject Heading**:	Ear, nose and throat/otolaryngology
Secondary Subject Heading:	Ear, nose and throat/otolaryngology, Epidemiology, Qualitative research
Keywords:	Paediatric otolaryngology < OTOLARYNGOLOGY, EPIDEMIOLOGIC STUDIES, QUALITATIVE RESEARCH

Protocol for the Paediatric Otorrhoea Study (POSt): a multi-methods study to understand the burden of paediatric otorrhoea in the UK

Elliot Heward[1], James Dempsey[2], Judith Lunn[3], John Molloy[1,2], Rachel Isba[3,4], Matthew Carr[5], Darren M Ashcroft[5,6], Alastair D Hay[7], Jaya R Nichani[1,2], Iain A Bruce[1,2]

[1]Division of Infection, Immunity and Respiratory Medicine, School of Biological Sciences, Faculty of Biology, Medicine and Health, University of Manchester, UK
[2]Royal Manchester Children's Hospital, Manchester University Hospitals NHS Foundation Trust, UK
[3]Lancaster Medical School, Lancaster University, Health Innovation One, Sir John Fisher Drive, Lancaster, UK
[4]Alder Hey Children's Hospital, Alder Hey Children's NHS Foundation Trust, UK
[5]Division of Pharmacy & Optometry, School of Health Sciences, Faculty of Biology, Medicine and Health, University of Manchester, UK
[6]NIHR Greater Manchester Patient Safety Research Collaboration (PSRC), University of Manchester, UK
[7]Centre for Academic Primary Care, Bristol Medical School: Population Health Sciences, University of Bristol, Bristol, UK

Corresponding Author: Mr Elliot Heward

Email Address: elliotheward@doctors.org.uk

Address: Paediatric Otolaryngology Research Office, Royal Manchester Children's Hospital, Manchester University Hospitals NHS Foundation Trust, UK

Telephone: 01612765606

Keywords: paediatric otolaryngology, epidemiologic studies, qualitative research

Word Count: 2652

For peer review only

Abstract

Introduction

Paediatric otorrhoea (PO) refers to the leakage of fluid through a perforation in the ear drum, resulting from an infection of the middle ear of a child or young person (CYP). PO frequently results in hearing loss which may lead to developmental delay, restricted communication and reduced educational attainment.

Epidemiological information for PO is largely derived from low-income countries. The aim of this study will be to establish the incidence of PO within the UK and to understand the impact of PO on CYP and their families' everyday lives. It will build the foundations for a randomised controlled trial investigating the best antibiotic treatment for PO.

Methods and Analysis

The study will consist of two work packages. 1) Data from the Clinical Practice Research Datalink (CPRD), January 2005 to July 2021, will be used to determine the incidence of patient presentations with PO to primary care in the UK. It will also explore the current antimicrobial prescribing practice for PO in primary care. 2) Thirty semi-structured interviews will be conducted from 13th July to 31st October 2023 with CYP and their parents/carers to help identify the impact of PO on everyday life, the patient journey and how service users define treatment success. Three medical professional focus groups will be used to understand the current management practice, how treatment success is measured and acceptability to randomise patients. Thematic analysis will be used.

Ethics and dissemination

The Health Research Authority, The Health and Social Care Research Ethics Committee (23/NI/0082) and the CPRD's research data governance panel (22_002508) reviewed this study. Results will be disseminated at medical conferences, in peer-reviewed journals and via social media. The study will co-create a webpage on healthtalk.org, with the Dipex Charity, about PO so to ensure members of the public can learn more about the condition.

For peer review only

Strengths and Limitations of the Study

- Patient and public involvement has been used to shape the study aims and design.
- The CPRD provides access to anonymised population-based electronic health records from general practices in the UK which will help determine the incidence and antibiotic treatment for paediatric otorrhoea.
- The limitation of CPRD database is that missing data is inevitable due to coding variation and incidence rates will be based solely on primary care presentations.
- Involvement of patients, their parents/carers and medical professionals will provide a range of experiences.
- The qualitative results will identify the most common themes and will not encompass all possible patient or medical professional experiences.

Introduction

Paediatric otorrhoea (PO) is discharge from a child's ear, that usually results from an infection of the middle ear. The accumulation of pus in the middle ear space causes the tympanic membrane to stretch until it ruptures, with the resultant leakage of foul-smelling discharge from the ear; called Acute Otitis Media with discharge (AOMd). If AOMd persists it can lead to a permanent perforation of the ear drum with chronic discharge. The World Health Organization (WHO) classifies otorrhoea lasting more than two weeks as Chronic Suppurative Otitis Media (CSOM), although various alternative time frames are used in the medical literature [1].

In routine clinical practice, classification systems for ear infections based on chronicity are not widely adopted by clinicians as they are not considered helpful in guiding management decisions. The presence, or absence, of otorrhoea being considered of greater relevance to treatment decisions. Our study group prefers to use the term Paediatric Otorrhoea (PO) to encompass the single disease process (AOMd and CSOM) as it translates more readily into clinical practice.

It is estimated that PO affects 50 million CYP per year globally [2-4]. PO may cause significant temporary or permanent morbidity in children, most frequently resulting in hearing loss, which may then lead to developmental delay, restricted communication, poor psychosocial development, and reduced educational attainment[1]. The WHO estimates that chronically discharging ears account for over half of hearing disability globally[1]. In addition, significant complications can occur, resulting in temporary or permanent sequelae such as imbalance, facial weakness and brain infections.

For peer review only - http://bmjopen.bmj.com/site/about/guidelines.xhtml

The psychosocial impact of PO on children and young people (CYP) and their care givers is poorly understood. Caregiver concern has been shown to be more prominent than the physical symptoms of PO in the limited published literature [5]. Public involvement (CYP and their carers) during development of this study was supported by a National Institute for Health and Care Research (NIHR) Research Design Service (RDS) public involvement grant (RDSNW3687). Experts-by-experience described years of recurrent ‘smelly ’discharge negatively impacting on education, sporting activities and socialisation. CYP that were affected, as well as carers, explained that educators and family members seemed to not understand the condition. Caregivers felt that more effective treatment and quicker referral to secondary care was required.

The aim of this study is to develop high-quality data to form the foundations for a randomised controlled trial (RCT) investigating the management options for PO. This will be achieved through the following objectives:

- determine the incidence and treatment for PO in the UK;
- improve the understanding of the impact of PO on CYP and carers to ensure clinically meaningful outcome measures are selected;
- understand how and why CYP with PO are currently treated and to assess treatment and randomisation acceptability for CYP, their carers and healthcare professionals for a future RCT.

Methods and Analysis

Study Design

This study will be divided into two work packages to answer the research objectives (Figure 1).

Patient and Public Involvement

This study has co-design and co-production running as a theme throughout. A (RDS) public involvement grant allowed the involvement of CYP with PO and their parents/carers during the study design stage. Their suggestions and experiences helped to shape every aspect of the study design. For example, parents/carers suggested either virtual meetings or phone calls were preferable to a face-to-face meeting due to their flexibility around childcare. This feedback is reflected in the study design.

A Patient Advisory Group (PAG) will be formed for the study, which will consist of 4-6 young people/parents/guardians who will meet at 3-to-4-monthly intervals. The goals

For peer review only - http://bmjopen.bmj.com/site/about/guidelines.xhtml

of the PAG are to help maintain the patient-focused nature of the work, maintain transparency, guide the design of the interview template and written patient information materials, as well as direct dissemination of the results.

To help determine the most effective methods of research dissemination and to help gain feedback on the design of a subsequent RCT, a study design workshop with public representatives will be conducted in January 2024. During the current study, we will identify and invite experts-by-experience to join the investigative team for the development of the subsequent research.

All members of the public will be supported by the PPI lead before, during and following the study. Training and support will be provided to all service users involved in this research.

Work Package 1

Design

Anonymised patient electronic health record data will be sourced from the CPRD. The CPRD contains electronic healthcare records from 1,760 (21% of all) general practices, including over 16 million patients, in the UK [6,7]. Of these patients, approximately 20% are aged under 18 years old [8]. Data will be utilised from the Aurum dataset.

During the study period of interest, 1st January 2005 to 31st July 2021, CYP aged 16 years and below will be included. Patients will be followed up until: index PO episode, death, date of transfer out of practice, last date of data collection, study end date or their 16th birthday, whichever comes earliest.

We will calculate annual incidence rates for PO categorised by: biological sex assigned at birth, age, geographical location and Index of Multiple Deprivation (IMD). We will generate these estimates by counting the number of cases and divide them by the total person-years at risk. The incidence will be presented as per 1000 person-years with its 95% confidence interval. Antibiotic prescription data (by route and type) will also be examined.

Outcomes

Primary outcome measure: diagnosis with paediatric otorrhoea

Secondary outcomes measures:

- type and frequency of antibiotic prescriptions for PO;
- frequency of referrals to secondary care ear, nose and throat services.

For peer review only - http://bmjopen.bmj.com/site/about/guidelines.xhtml

Factors measured:

- biological Sex at Birth
- age
- geographical location
- index of Multiple Deprivation

Sample Size

The proposed study is descriptive in design, therefore we do not have specific hypotheses and a formal sample size calculation is not required.

Limitations

The major limitations of the database are missing data and coding variation. Incomplete diagnoses, or those coded using free text, will not be identified by the CPRD. To ensure the correct patient presentations were identified an exhaustive list of codes related to PO was generated for the Aurum database (supplementary file). Four clinicians reviewed the coding list. Codes were categorised as primary codes (correctly identify patients with otorrhoea), secondary codes (may identify patients with otorrhoea) or irrelevant coding. The secondary list will be used for a sensitivity analysis. Where clinicians disagreed in the coding decision, a consensus was reached following discussion. Codes were classified as secondary codes if there was no majority consensus to whether the code described PO. Codes relating to grommets or tympanostomy tubes were excluded as infection of grommets can also cause otorrhoea. Missing outcome data are not likely to be a major issue since diagnostic codes are binary and therefore indicate the presence or absence of clinical condition.

Analysis

Crude overall incidence rates (with 95% confidence intervals) will be estimated. We will also produce internally age- and sex-standardised incidence rates over the duration of the study period. The data will then be structured in an annual time-series format with event counts and ‘person-years at risk’ aggregated (by year) with stratification by sex, age group and deprivation (IMD) quintile. Mean-dispersion negative binomial regression models will be used to describe the trends in incidence rates over time. The natural logarithm of the denominator (person-years at risk) will be used as an offset in each regression model. To account for potential non-linear trends, calendar time will be modelled using splines or fractional polynomials. Practice-level variation will be examined using multi-level variants of the regression models with practice modelled using random effects. Sensitivity analyses will be

For peer review only - http://bmjopen.bmj.com/site/about/guidelines.xhtml

undertaken to compare the incidence rates using the primary and secondary coding lists only.

Work Package 2

Study Design

Semi-structured interviews will be conducted with CYP and their parents/carers, and focus groups with medical professionals. A semi-structured interview approach has been chosen due to its interactive and flexible nature, which is useful to capture the reality of everyday experience. The interview guide for the interviews and focus groups are expected to evolve following participant responses (supplementary file). Inclusion criteria is outlined in Table 1.

Sample Size

The recruitment aim for the patient interviews is 30 interviews or when data saturation is reached and the minimum number of patients with key demographics have participated (Table 2). The recruitment aim for the medical professional focus groups is 24 participants divided over three focus groups. The sample size is estimated to allow for sufficient representation of the population of patients with PO. The study aims to capture a wide variety of experiences and practices within the medical professional cohort. Prior research studies addressing similar clinical questions and population were considered to reach the proposed sample size [9]. It also takes into account the cost and time required to undertake 30 patient interviews and three focus groups.

Recruitment

Patient Interviews

Patient recruitment will use a direct-to-patient method by advertising the study using posters in medical establishments (e.g. GP practices and hospital outpatient waiting rooms), online (e.g. charities, societies and institutions) and on social media.

The poster advertisement will direct the patient or carer to contact the research team by text, call or email. Recruitment will take place via a single institution. After the patient contacts the research team and expresses an interest in participating the cover letter, parent or carer information sheet and age-appropriate patient information sheet (PIS) will be sent to the participant.

For peer review only - http://bmjopen.bmj.com/site/about/guidelines.xhtml

The patient and parent/carer will be given the opportunity to read the study material to determine if they would like to participate. There will be a minimum of two days between sending the information material to the patient and arranging an interview.

In acknowledgement of participants' valuable time, they will be remunerated with a gift £25 voucher in line with the NIHR guidelines for public involvement renumeration [10]. Participants will be offered to be informed of the research findings.

Medical Professional Focus Groups

Recruitment will use a direct-to-participant advertisement. A poster advertising the study will be sent for distribution through professional bodies, medical institutions and to primary and secondary care settings. The study poster will direct medical professionals to contact the research team to take part. Once they contact the research team, they will be sent the medical professional cover letter and PIS and booked onto a focus group session.

All participants will be offered to be informed of the research findings and will be acknowledged individually in publications produced from this work.

Consent

Informed consent will be obtained prior to the participants undergoing any study related activities. The qualitative researcher will lead the consent discussion with all participants and will have up-to-date GCP training.

To improve dissemination of the research findings, the study team is working with the Dipex charity to create a webpage on https://healthtalk.org where information about PO can be shared publicly. The qualitative researcher will invite patients (CYP and their parents/carers) to be involved by contributing short clips of their video recording, audio recording or a transcript of their interview to the web page. Participating in this aspect of the study is optional and does not preclude the patients from taking part in the study.

Data Collection

Patient interviews

Interviews are expected to last between 45-60 minutes, but the length of the interview will be guided by the participant. The patient's age at the time of interview and sex will be collected from the patient or parent/carer.

For peer review only - http://bmjopen.bmj.com/site/about/guidelines.xhtml

Parents/carers will be invited to take part in the interview on behalf of children younger than 5 years old. CYP who are 5 years or older will be invited to participate in joint interviews with their parents/carers, to recognise the importance of their experiences.

The study aims to reduce barriers to participation by using interpreters in the study. Sign language interpreters will be available for virtual or face-to-face interviews if required. An interpreter will be used for non-English-speaking participants. The interpreter will be used for patient consent and interview. Written material will be translated into any language for up to 6 participants if required. This number is based on the proportion of non-English speaking people in England and Wales. Translation will be performed depending on patient preference. Where possible, to improve the interview dynamic, the interpreter and the interviewer will be in the same location if a virtual interview is taking place. Interviews involving an interpreter will be allocated additional time to allow for translation time.

Medical Professional Focus Groups

Semi-structured focus groups will be conducted with groups consisting of 6-8 participants. Nurses, audiologists, allied medical professionals, GPs, Emergency Department and Otolaryngology doctors will be invited to participate. Healthcare professionals from varied demographic locations and of different grades will be invited to take part in the study. Focus groups will last for up to 60 minutes.

Data Analysis

Audio recording will be transcribed verbatim. The anonymised transcriptions will be imported to qualitative data analysis software for data management. Thematic analysis will follow guidance provided by Braun and Clarke (2006) who suggest the five key stages of thematic analysis include: (1) familiarizing with the data (2) generating codes (3) identifying themes (4) reviewing themes and collating into a thematic map or matrix (5) write up the findings [11]. We will also refer to Braun and Clarke's (2021) recent assessment guidelines for evaluating thematic analysis research quality to ensure a well-developed and justified analysis, and to avoid the most common problems with this approach ('proceduralism' or a non-reflexive grouping of themes) [12]. There will be multiple independent coders drawn from the research team, as well as inter-rater reliability measures applied to the iterative process from codes to themes [11,13]. The researchers will read and re-read the transcript to familiarise themselves with the data. Codes will then be generated in a systematic manner across the data set. Subsequently, the codes will be collated into potential themes which will then be reviewed and refined. Two researchers will independently identify themes before discussion in order to reach consensus.

For peer review only - http://bmjopen.bmj.com/site/about/guidelines.xhtml

Ethics and Dissemination

The Clinical Practice Research Datalink (CRPD) protocol was approved by the CPRD's research data governance process (*22_002508*) on 4 April 2023. The qualitative study was approved by The Health Research Authority on 23 June 2023 and The Research Ethics Committee (REC 23/NI/0082) on 15 June 2023.

This study will work with PAG members, service users involved in the study workshop and the Dipex charity to formulate an engagement plan to ensure the research findings are heard by the relevant audience. We will develop online public resources on healthtalk.org with the Dipex charity about PO. We aim to empower PAG members to help disseminate the research outcomes via local networks and online forums. Results will be disseminated to medical professionals at conferences, in peer-reviewed journals and via social media.

Authors Contributions

Study conception and overall study design (EH & IB). Design of the public engagement elements (JD & JN). Design of work package 1 (MC, DA & AH). Design of work package 2 (JL, RI & JM). EH and JD drafted the manuscript which was reviewed by all authors.

Funding statement

This work was supported by the National Institute of Health and Care Research (NIHR) grant number NIHR204036. DMA is supported by the NIHR Greater Manchester Patient Safety Research Collaboration (PSRC).

Competing Interests

No completing interests declared.

References

1. World Health Organization. Chronic suppurative otitis media. Burden of illness and management options. Available: https://apps.who.int/iris/handle/10665/42941 [Accessed 22 July 2023]
2. Little P, Gould C, Williamson I, *et al*. Pragmatic randomised controlled trial of two prescribing strategies for childhood acute otitis media. BMJ 2001;10:336-42. doi: 10.1136/bmj.322.7282.336

For peer review only

3. Monasta L, Ronfani L, Marchetti F, *et al*. Burden of disease caused by otitis media: systematic review and global estimates. PLoS One. 2012;7:e36226. doi: 10.1371/journal.pone.0036226

4. Amnesty International. Children's Human Rights. Available: https://www.amnesty.org/en/what-we-do/child-rights/ [Accessed 22 July 2023]

5. Vlastos IM, Kandiloros D, Manolopoulos L, *et el*. Quality of life in children with chronic suppurative otitis media with or without cholesteatoma. Int J Pediatr Otorhinolaryngol. 2009;73:363-9. doi: 10.1016/j.ijporl.2008.10.030

6. Clinical Practice Research Datalink. (2022). CPRD Aurum March 2022 (Version 2022.03.001) [Data set]. Clinical Practice Research Datalink. https://doi.org/10.48329/MY9S-4X08

7. Clinical Practice Research Datalink. (2022). CPRD GOLD March 2022 (Version 2022.03.001) [Data set]. Clinical Practice Research Datalink. https://doi.org/10.48329/5RHX-ER29

8. Wolf A, Dedman D, Campbell J, *et al*. Data resource profile: Clinical Practice Research Datalink (CPRD) Aurum. Int J Epidemiol. 2019;48:1740-1740g. doi: 10.1093/ije/dyz034

9. Vasileiou K, Barnett J, Thorpe S, *et al*. Characterising and justifying sample size sufficiency in interview-based studies: systematic analysis of qualitative health research over a 15-year period. BMC Med Res Methodol. 2018;18:148. doi: 10.1186/s12874-018-0594-7

10. NIHR. Payment guidance for researchers and professionals. Available: https://www.nihr.ac.uk/documents/payment-guidance-for-researchers-and-professionals/27392 [Accessed 22 July 2023]

11. Braun V, Clarke V. Using thematic analysis in psychology. Qualitative research in psychology. 2006;3:77-101. Doi: 10.1191/1478088706qp063oa

12. Braun V, Clarke V. Thematic Analysis: A Practical Guide. Sage publications, 2021

13. Braun V, Clarke V. One size fits all? What counts as quality practice in (reflexive) thematic analysis?. Qualitative research in psychology. 2021;18:328-52. Doi: 10.1080/14780887.2020.1769238

For peer review only - http://bmjopen.bmj.com/site/about/guidelines.xhtml

Tables

Table 1. Inclusion and Exclusion Criteria

**Inclusion Criteria**	
Patient Interviews	• Children and young people aged 16 years and under<br>• Children and young people who have had otorrhoea in the past year<br>• Caregiver legally able to give consent for the CYP or the CYP themselves has the capacity to consent<br>• Live in the United Kingdom (UK)<br>• All languages (up to six non-English-speaking participants)
Medical Professional Focus Groups	• Medical professionals with experience of caring for CYP with otorrhoea in their clinical practice<br>• Currently practicing in the UK<br>• English-speaking

Table 2. Minimum Patient Recruitment Numbers by Demographic

**Demographic**		**Minimum Number of Patients**
Age	0-4 years	3
	5-8 years	3
	9-12 years	3
	13-16 years	3
Sex	Male	5
	Female	5
Disease Presence	Active otorrhoea	4
	Previous otorrhoea within last year	4

Figure Legends

Figure 1. Study work packages and linked study objectives (PO = paediatric otorrhoea)

For peer review only - http://bmjopen.bmj.com/site/about/guidelines.xhtml
For peer review only

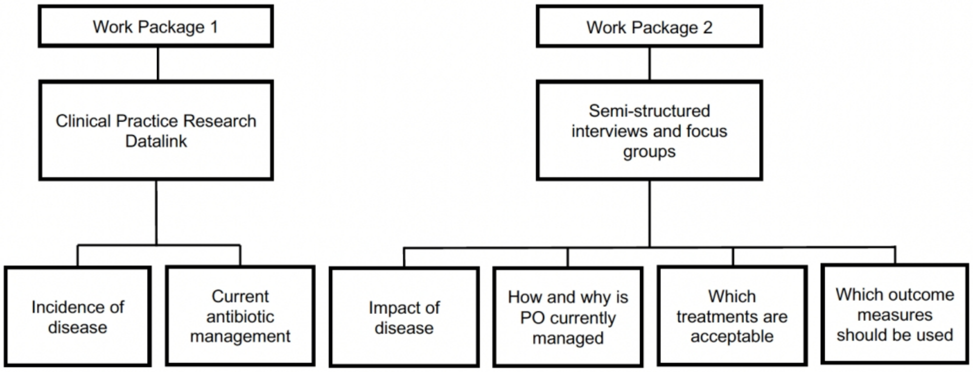

Figure 1. Study work packages and linked study objectives (PO = paediatric otorrhoea).

280x110mm (144 x 144 DPI)

medcodeid	readcode	snomedctconceptid	snomedctdescriptionid	Aurum Database	Coding Selection
8.45868E+15		1.08256E+15	3078540019	Recurrent acute suppurative otitis media with spontaneous rupture of ear drum	Primary
253190015	1C4Z.00	162362000	253183013	Ear discharge symptom NOS	Primary
253183013	1C4..00	162362000	253183013	Ear discharge symptoms	Primary
4.5481E+15		162364004	253187014	Discharge from ear	Primary
4.54811E+15		162364004	253188016	Discharging ear	Primary
253186017	1C42.00	162364004	253186017	Ear discharge present	Primary
255616012	2D6..00	164211000	255616012	O/E - discharge from ear	Primary
255623013	2D6Z.00	164211000	255616012	O/E - ear discharge NOS	Primary
4.57677E+15		164211000	2667264011	On examination - discharge from ear	Primary
255618013	2D62.00	164213002	255618013	O/E - serous ear discharge	Primary
4.57681E+15		164213002	2667266013	On examination - serous ear discharge	Primary
255620011	2D64.00	164215009	255620011	O/E - purulent ear discharge	Primary
4.57685E+15		164215009	2666962013	On examination - purulent ear discharge	Primary
5.8754E+15		300132001	1490583015	Discharge from ear	Primary
5.87537E+15		300132001	1477433018	Ear discharge	Primary
5.87538E+15		300132001	441141019	Finding of ear discharge	Primary
5.87539E+15		300132001	1226738019	Observation of ear discharge	Primary
3.06032E+15		34790005	58064015	Benign chronic suppurative otitis media with anterior perforation of ear drum	Primary
3.11845E+15		38394007	490747016	CSOM - Chronic suppurative otitis media	Primary
299067013	F523.00	38394007	63479018	Chronic suppurative otitis media	Primary
299529015	FyuP200	38394007	63478014	[X]Other chronic suppurative otitis media	Primary
6.22921E+14	F586011	65668001	109100010	Discharge of ear	Primary
3.56778E+15		65668001	109101014	Drainage from external ear canal	Primary
3.56776E+15		65668001	109096014	Otorrhea	Primary
109102019	F586.00	65668001	109102019	Otorrhoea	Primary
8.83841E+14	F586.99	65668001	8.83841E+14	Otorrhoea - discharging ear	Primary

For peer review only

299229016	F586z00	65668001	109102019	Otorrhoea NOS	Primary
399506010	F586000	65668001	109102019	Unspecified otorrhoea	Primary
1.27288E+16		6.94131E+14	1.51841E+15	Chronic suppurative otitis media NOS	Primary
4.60551E+14	F520100	86279000	1235118013	Acute suppurative otitis media - tympanic membrane ruptured	Primary
3.90297E+15		86279000	1235119017	Acute suppurative otitis media with discharge	Primary
3.90295E+15		86279000	143085014	Acute suppurative otitis media with spontaneous rupture of ear drum	Primary
5.56821E+14	F521.00	87665008	1235262012	Chronic suppurative otitis media - tubotympanic	Primary
3.92483E+15		87665008	145352014	Chronic tubotympanic suppurative otitis media	Primary
4.06248E+15		95803004	158677012	Serous drainage from external ear canal	Primary
4.06249E+15		95804005	158678019	Purulent drainage from external ear canal	Primary
4.06251E+15		95805006	158679010	Foul odor drainage from external ear canal	Primary
4.0625E+15		95805006	201783016	Foul odour drainage from external ear canal	Primary
3.11848E+15		38394007	490751019	Chronic otitis media with perforation	Secondary
3.17615E+15		41954005	70007011	Chronic atticoantral suppurative otitis media	Secondary
5.56811E+14	F522.00	41954005	1229812011	Chronic suppurative otitis media - atticoantral	Secondary
4.77469E+15		194281003	299062019	ASOM - Acute suppurative otitis media	Secondary
4.7747E+15		194281003	299064018	Acute purulent otitis media	Secondary
299061014	F520.00	194281003	299061014	Acute suppurative otitis media	Secondary
299066016	F520z00	194281003	299061014	Acute suppurative otitis media NOS	Secondary
4.77471E+15		194282005	2575709017	Acute suppurative otitis media due to another disease	Secondary
299065017	F520300	194282005	299065017	Acute suppurative otitis media due to disease EC	Secondary
299069011	F524000	194286008	299069011	Bilateral suppurative otitis media	Secondary
3.06031E+15		34790005	58063014	Chronic tubotympanic disease with anterior perforation of ear drum	Secondary
490746013	F513100	38394007	490746013	Chronic otitis media with effusion, purulent	Secondary
8.83741E+14	F523.99	6.94131E+14	8.83741E+14	Chronic purulent otitis media	Secondary
490749018	F513111	38394007	490749018	Chronic secretory otitis media, purulent	Secondary
3.11847E+15		38394007	490750018	Otitis media with effusion - purulent	Secondary

For peer review only

1.16801E+14	F52..00	39288006	65880011	Purulent otitis media	Secondary
299068015	F524.00	39288006	65880011	Purulent otitis media NOS	Secondary
3.13204E+15		39288006	65883013	Suppurative otitis media	Secondary
3.17617E+15		41954005	70011017	Persistent mucosal disease with posterior AND/OR superior marginal perforation of ear drum	Secondary
8.83741E+14	F523.99	6.94131E+14	8.83741E+14	Chronic purulent otitis media	Secondary
7.98739E+15		7.361E+12	3022971014	Perforation of tympanic membrane due to otitis media	Secondary
504727012	F511.11	81564005	504727012	Chronic secretory otitis media, serous	Secondary
135302019	F511.99	81564005	8.83691E+14	Chronic serous otitis media	Secondary
299024017	F511z00	81564005	135302019	Chronic serous otitis media	Secondary
3.82626E+15		81564005	504725016	Glue ear - serous	Secondary
5.55791E+14	F511.00	81564005	504726015	Otitis media with effusion - serous	Secondary
3.82625E+15		81564005	135305017	Simple chronic serous otitis media	Secondary
299023011	F511300	81564005	135302019	Unilateral chronic serous otitis	Secondary
5.0338E+15		232251007	347975014	Recurrent acute suppurative otitis media	Secondary
6.01386E+15		312218008	455892014	Infective otitis media	Secondary
11759018	F512100	6485001	11759018	Mucosanguinous chronic otitis media	Secondary
7.57612E+15		703469002	3008656010	Bacterial otitis media	Secondary
7.82221E+15		721742004	3325908013	Otitis media caused by Streptococcus pneumoniae	Secondary
7.82222E+15		721742004	3325909017	Streptococcus pneumoniae otitis media	Secondary

For peer review only

POSt Patient Interview Guide

Welcome and introduction - Seek verbal consent to continue, reminder of audio-recording of interview (or video recording if on Microsoft Teams), re-cap of project and plan for interview.

The study is aiming to understand what it is like for children and young people to live with an ear infection which causes a leaky ear. The interviews will explore your views and we are interested in your experience. It will help us to understand more about children and families' experiences of leaky ears. We also wanted to talk to you about how we might conduct research in the future on leaky ears, and if you think it is acceptable way of doing things. With this information we hope to help change how we treat children and young people with leaky ears.

If you do not feel you are able to comment on any area, please say so and we can stop at any time. Do you have any questions before we start?

1. Patient and carer experience of the condition

2. Patient and carer experience of treatment

3. Identifying in the patient and carer's opinion what is the best sign to use for treatment success

Lay explanation of randomised controlled trial is given by facilitator.

4. Identifying in the patient and carer's opinion on randomisation in a future trial

5. Identifying in the patient and carer's opinion on taking placebo medications

6. Identifying in the patient and carer's opinion on motivators and barriers to participating in a randomised controlled trial

Anything not covered?

Is there anything that we haven't covered in the interview that you think we should know or think about?

Closing and thanks - Thank for their time and contribution.

For peer review only

POSt Medical Professional Interview Guide

Welcome and introduction - Seek verbal consent to continue, and check that all members must interact with patients aged 16 and below with paediatric otorrhoea in daily practice to be eligible to take part. Provide a reminder of the video-recording of focus group (Microsoft Teams) will be saved, re-cap of project and plan for focus group discussion. Perform member check after each question or where necessary.

The study is aiming to understand how and why children and young people with PO are treated in primary and secondary care and to understand what treatments are acceptable to medical professionals. We are interested in your perceptions, based on your knowledge and experience. If you do not feel you are able to comment on any area, please say so. Do you have any questions before we start?

1. Introduction of group members and job role

2. Experiences of managing children and young people with paediatric otorrhoea

3. Normal treatment practices

4. Identifying treatment success

5. Motivations and barriers to participating in a future randomised controlled trial

Anything not covered?

Is there anything that we haven't covered in the focus group that you think we should know or think about?

Closing and thanks - Thank for their time and contribution.

For peer review only
