# Supplementary material for: Protocol for the Paediatric Otorrhoea Study (POSt): a multi-methods study to understand the burden of paediatric otorrhoea in the UK
Source: BMJ Open. 2023 Sep 5;13(9):e078052. doi: 10.1136/bmjopen-2023-078052 (PMC10481712; doi:10.1136/bmjopen-2023-078052)
Supplement: Reviewer comments [file bmjopen-2023-078052.reviewer_comments.pdf]

PEER REVIEW HISTORY

BMJ Open publishes all reviews undertaken for accepted manuscripts. Reviewers are asked to complete a checklist review form (**http://bmjopen.bmj.com/site/about/resources/checklist.pdf**) and are provided with free text boxes to elaborate on their assessment. These free text comments are reproduced below.

ARTICLE DETAILS

**TITLE (PROVISIONAL)**	Protocol for the Paediatric Otorrhoea Study (POSt): a multi-methods study to understand the burden of paediatric otorrhoea in the UK
**AUTHORS**	Heward, Elliot; Dempsey, James; Lunn, Judith; Molloy, John; Isba, Rachel; Carr, Matthew; Ashcroft, Darren; Hay, Alastair; Nichani, Jaya; Bruce, Iain

This article was not externally reviewed at BMJ Open. Protocols that have been independently assessed prior to submission to BMJ Open are usually fast-tracked to publication on the grounds that further substantial changes will not be possible. This independent assessment will usually be external, independent review for both a substantial grant award from a non-commercial or government funder and ethics approval.
